# Differential sensitivities to blood pressure variations in internal carotid and intracranial arteries: a numerical approach to stroke prediction

**DOI:** 10.1038/s41598-023-49591-3

**Published:** 2023-12-15

**Authors:** Muhsin Kizhisseri, Saleh Gharaie, Sethu Raman Boopathy, Ruth P. Lim, Milad Mohammadzadeh, Jorg Schluter

**Affiliations:** 1 School of Engineering, Deakin University, 75 Pigdons Rd, Waurn Ponds, VIC 3216 Australia; 2 See-Mode Technologies Pte Ltd, Melbourne, Australia; 3 Austin Health, Melbourne, Australia

**Keywords:** Atherosclerosis, Fluid dynamics

## Abstract

Stroke remains a global health concern, necessitating early prediction for effective management. Atherosclerosis-induced internal carotid and intra cranial stenosis contributes significantly to stroke risk. This study explores the relationship between blood pressure and stroke prediction, focusing on internal carotid artery (ICA) branches: middle cerebral artery (MCA), anterior cerebral artery (ACA), and their role in hemodynamics. Computational fluid dynamics (CFD) informed by the Windkessel model were employed to simulate patient-specific ICA models with introduced stenosis. Central to our investigation is the impact of stenosis on blood pressure, flow velocity, and flow rate across these branches, incorporating Fractional Flow Reserve (FFR) analysis. Results highlight differential sensitivities to blood pressure variations, with M1 branch showing high sensitivity, ACA moderate, and M2 minimal. Comparing blood pressure fluctuations between ICA and MCA revealed heightened sensitivity to potential reverse flow compared to ICA and ACA comparisons, emphasizing MCA's role. Blood flow adjustments due to stenosis demonstrated intricate compensatory mechanisms. FFR emerged as a robust predictor of stenosis severity, particularly in the M2 branch. In conclusion, this study provides comprehensive insights into hemodynamic complexities within major intracranial arteries, elucidating the significance of blood pressure variations, flow attributes, and FFR in stenosis contexts. Subject-specific data integration enhances model reliability, aiding stroke risk assessment and advancing cerebrovascular disease understanding.

## Introduction

Ischemic stroke (IS), a subtype of stroke characterized by brain ischemia due to thrombosis of a cerebral blood vessel, is a leading neurovascular cause of death and disability. Between 1990 and 2019, the global number of IS deaths increased from 2.04 million to 3.29 million, and it is projected to rise further to 4.90 million by 2030^1^. Atherosclerosis, a pathological condition marked by the accumulation of lipids and fibrous elements within arterial walls, plays a significant role in the onset of ischemic stroke. The internal carotid artery (ICA) which supply blood from the heart to brain through Middle Cerebral Artery (MCA) and Anterior Cerebral Artery (ACA) is particularly vulnerable to atherosclerosis.

In recent years, there has been a growing interest in the use of the systolic and diastolic blood pressure drop measurements measured using auscultatory techniques and Fractional Flow Reserve (FFR) as the biomarkers for stroke prediction as they are key indicators of the severity of atherosclerosis leading to carotid and intra cranial stenosis^2–4^. The progression of atherosclerosis eventually resulting in the formation of the plaque structures which changes the blood flow patterns in the location leading to changes in the blood pressure. Initially, fatty streaks form on arterial walls composed of cholesterol and immune cells, causing slight narrowing and minimal impact on blood pressure. Over time, these streaks develop into more advanced plaques with a lipid core and fibrous cap, further narrowing arteries and raising resistance, potentially elevating blood pressure. In advanced stages, plaque rupture exposes the core, prompting blood clot formation, which can partially or fully block arteries, causing the insufficient blood supply into brain and severe outcomes like strokes^5–9^. To better understand the relationship between blood pressure and stroke, researchers have increasingly turned to computational fluid dynamics (CFD) substantiated by the Windkessel model-derived outlet boundary conditions^3,10,11^. Precise measurement of intravascular pressure in carotid arteries is also very important in understanding cerebrovascular mechanics, particularly in the case of diseased patients, as pressure plays a significant role alongside wall shear stress in understanding the hemodynamics of blood flow with in the cerebral arteries^12–14^. Studies have reported the importance of measuring the central systolic pressure in arteries such as ascending aorta for the analysis of cardio-related disease and have shown that it can improve the prediction of mortality^15^. Waddell et al. reported that carotid arterial blood pressure is the better predictor to assess the severity of cardiovascular and cerebrovascular diseases compared to brachial arterial blood pressure which further directs towards the importance of the blood pressure measurement in the internal carotid arteries and intracranial arteries^16^.

One of the key challenges in studying FFR as biomarkers for stroke prediction is the difficulty in accurately measuring these variables in vivo. Currently, stroke neurologist uses various invasive methods such as Invasive Arterial Catheterization to measure the FFR inside the cerebral artery which carries the risks of infection, haemorrhage and other complications to patient depends on their medical conditions^17–21^. The non-invasive techniques such as Transcranial Doppler (TCD) ultrasound technique can measure the blood flow velocity in cerebral arteries, but it has major limitations such as limited spatial resolution and heavy operator dependence, requiring in-depth knowledge of cerebrovascular anatomy, and a 10–15% rate of inadequate acoustic windows may stem from bone thickness around acoustic windows, affecting ultrasound transmission. TCD can only measure large basal arteries and offers a global cerebral blood flow velocity index, lacking local blood flow information^22,23^. The Clinical imaging techniques such as magnetic resonance imaging (MRI) and computed tomography (CT) are non-invasive but they have limited spatial resolution and does not provide real-time monitoring, making it difficult to accurately measure blood pressure in small vessels and in specific regions of the brain. However, the use of CFD can overcome these challenges by providing a non-invasive method for predicting blood pressure in the cerebrovascular system using patient-specific anatomic data from non-invasive imaging modalities. CFD stands out as a reliable, non-invasive, and cost-effective method for understanding complex arterial regions like the ICA, ACA and MCA. It provides accurate approximations of velocity, pressure, and wall shear stress distributions in a 3D visual representation that's hard to achieve non-invasively^24–30^. CFD simulations offer insight into intricate and inaccessible regions with high spatial and temporal resolution, offering a better alternative to costly and time-consuming physical diagnostics tools. These simulations have demonstrated strong agreement with in vivo results, establishing CFD as a valuable tool in studying hemodynamics^26,31–33^.

Complicated and disrupted blood flow occurs in regions of intricate cardiovascular geometry, notably curvatures and bifurcations^34^. The structure and form of carotid arteries were scrutinized through CT angiography, revealing the intricate geometry stemming from the Common Carotid Artery bifurcation. This intricate geometry fosters regions of pronounced blood flow disturbance, rendering them susceptible to heightened atherosclerotic risk, potentially leading to stenosis^35^. The carotid bulb emerges as a pivotal site of atherosclerotic vulnerability, harbouring regions characterized by perturbed flow, spanning both healthy and pathological carotid states^36^. An investigation disclosed that 80% of strokes originate from stenosed carotid arteries^37^, underscoring the salience of hemodynamic carotid artery analysis in the context of stroke prediction. Drawing from a numerical study, Umberto et al. selected the carotid artery to delve into outlet boundary condition influence on atherosclerosis, owing to the internal carotid artery's ability to mirror the intricate interplay of hemodynamic parameters within blood flow and factors governing vessel wall pathology^38^. The Internal Carotid Artery (ICA) branches into the Middle Cerebral Artery (MCA) and Anterior Cerebral Artery (ACA), yielding perturbed flow within the ICA's bifurcation zones. Further bifurcation sees MCA divide into segments—M1, M2, M3, and M4—and ACA into segments A1, A2, A3, A4, and A5. To date, no study has delved into analysing the pivotal terminal ICA branches' influence on ICA hemodynamics across diverse locations. Moreover, the effects of stenosis in the segments of MCA and ACA on ICA blood flow distribution remain unexplored.

The significance of peripheral resistance within the cerebrovascular system is noteworthy, exerting substantial influence overflow partitioning at bifurcation points. Moreover, downstream resistance considerably shapes pressure and flow patterns within arteries^39^. Hemodynamic attributes like blood flow rate, viscosity, and vascular compliance are likewise subject to distal vascular resistance. Elevated distal resistance curtails arterial blood flow, prompting arterial dilation. Lee et al. found a 5% expansion in low wall shear stress areas during the systolic phase when distal vascular resistance increased by 20%^40^. A transient blood flow simulation at a carotid bifurcation underscores how elevated distal vascular resistance leads to reduced wall shear stress zones in the carotid artery, fostering atherosclerosis progression^38^.

Arterial stenosis contributes to augmented axial blood flow velocity due to diminished cross-sectional area within the artery^41^. The constriction of the carotid artery, arising from severe stenosis, not only lessens the artery's rigidity (due to heightened pressure in that locale) but also encourages greater arterial deformations^42^. Pivotal asymmetrical stenoses engender critical flow dynamics, precipitating arterial collapse and rupture of plaque caps and blood clots. Asymmetric stenosis can also induce a substantial 50% alteration in maximum shear stress and 30% shift in back pressure^43^. Additionally, sudden area shifts within the carotid artery induced by stenosis or variations in geometry can usher in flow separation. This, in turn, fosters regions of negative velocity or re-circulation, occasioning low wall shear stress (WSS) and inciting atherosclerosis^44^. Furthermore, at cerebral artery bifurcations, diminished shear rates prompt increased blood viscosity due to non-Newtonian shear-thinning behaviour, culminating in lowered cardiac output and elevated distal resistance, heightening atherosclerosis susceptibility^40^.

The carotid artery segment exhibiting maximum stenosis, combined with heightened wall shear stress (WSS), emerges as the primary locus for atherosclerotic plaque rupture^42^. Arterial stenosis also fosters the creation of recirculation zones downstream of constriction areas, induced by shifts in blood pressure^41^. Mancini et al. observed vortex formation in carotid artery models due to stenosis-triggered blood flow acceleration and deceleration, with these vortices extending downstream of stenotic segments^45^. Computational fluid dynamics (CFD) simulations on consecutively stenosed elastic arteries reveal that blood flow diverts toward the central axis post initial stenosis impact, culminating in a region of low shear stress at the second stenosis site. This arrangement engenders a high oscillatory area after the second stenosis, enlarging the atherosclerosis-prone domain^46^.

In recent years, the utilization of blood pressure measurements has ushered in a groundbreaking concept termed Fractional Flow Reserve (FFR), poised to supplant the conventional practice of assessing luminal diameter constraints. This traditional approach, marred by its inability to consider intricate hemodynamic effects, has demonstrated inadequacy in appraising stroke risk. Fractional Flow Reserve (FFR) denotes the ratio between maximum blood flow distal to a stenotic lesion during hyperemia, relative to the maximum flow in a healthy coronary vessel, induced by the administration of adenosine to induce coronary dilation. Revered as the gold standard for gauging the hemodynamic significance of vascular stenosis in cardiovascular studies, FFR has emerged as an invaluable tool in guiding patient selection for percutaneous coronary interventions, pinpointing ischemic regions^47^. However, in measuring Intracranial Cranial Atherosclerotic Stenosis (ICAS) using FFR technique, the major challenge arises as the inability of the intracranial arteries to achieve maximal vasodilation akin to their coronary counterparts. Consequently, this distinction has prompted research efforts to explore alternative markers not reliant on adenosine for characterizing the hemodynamic correlation of coronary artery stenosis^48^. Notably, these investigations have revealed a robust linear relationship between pre and post stenotic pressure ratio representing the pressure differential across the stenotic region^49^.

In recent years, the integration of Fluid–Structure Interaction (FSI) studies into Computational Fluid Dynamics (CFD) models has gained prominence, particularly in the context of arterial wall flexibility^2,50–58^. While many computational studies employ rigid wall assumptions, acknowledging the minimal impact of wall motion on blood pressure measurements, it is essential to recognize the increased sensitivity of wall shear stress (WSS) to wall deformations. Although pressure drops are less affected, FSI studies become crucial for accurate wall shear stress analysis, as demonstrated by various researchers^33,50,59–62^. Studies have shown that assuming rigid vessel walls can lead to significant overestimation of hemodynamic parameters like wall shear stress^50,63^. Notably, variations in wall thickness have been found to be critical in predicting accurate flow patterns, emphasizing the importance of incorporating FSI in intracranial hemodynamics studies^50^. Despite the high computational cost associated with FSI, studies focusing on pressure drop often adhere to rigid wall assumptions due to minimal variations in blood pressure measurements^61,64^.

In this study, a computational fluid dynamics approach is used to simulate blood flow in a three-dimensional geometric model of a subject specific ICA to identify the most influential branches of the ICA among the ACA, M1, and M2. This investigation centers on their role in determining hemodynamic attributes such as blood pressure, blood flow velocity, and blood flow rate at various points across the ICA, ACA, and MCA. To achieve this, stenosis into proximal sections of the ACA, M1, and M2 was introduced using the Windkessel model's resistance concept. Furthermore, this study integrated FFR analysis to evaluate the influence of stenosis within branches like M1, M2, and ACA on blood pressure distribution across the ICA and its associated branches.

## Methodology

The first objective of this study was to validate the Computational Fluid Dynamics (CFD) model by comparing its outcomes with clinical findings. A subject-specific, healthy internal carotid artery was employed for the study, with the geometry and flow measurement data provided by See-Mode Technologies Pte Ltd. The 3D Phase-Contrast Magnetic Resonance Imaging (PCMRI) data from 3T MRI machine, presenting blood flow velocity distributions within the ICA, MCA, and ACA, were utilized. The PCMRI data was averaged from various planes at diverse locations within the ICA, MCA, and ACA, all based on the subject's individualized geometry (Fig. 3). As validation was the foremost goal, the CFD model was meticulously aligned with PCMRI outcomes, employing extensive subject-specific data. ANSYS FLUENT, a commercial CFD software package, was used for simulations, incorporating the 3D subject-specific geometry. The clinical blood flow velocity at the ICA, derived from PCMRI data, served as the inlet boundary condition. A User-Defined Function (UDF) was generated according to the subject-specific velocity profile. Since the inlet velocity data wasn't precisely from the inlet of the subject's geometry, it was scaled to match the PCMRI velocity at the specific ICA location. This ensured the subject-specific velocity profile's integration into the CFD domain. The ACA and MCA also had blood velocity profiles from the PCMRI data. The CFD model was considered fully validated if its outcomes at certain MCA and ACA locations matched PCMRI results at those same spots. The validation process involved introducing distal resistances to ICA branches (Ophthalmic Artery (OA), ACA, M1, and M2), iteratively adjusted to align CFD outcomes with clinical findings. Iterations were performed systematically, tweaking resistance at each branch outlet until congruence was achieved between CFD and clinical results. Since the study's clinical data pertained to a healthy internal carotid artery, the final iteration's distal resistance values for ACA, M1, M2, and OA were deemed applicable to healthy cases. The initial values for distal resistance in outlets such as the OA, ACA, M1, and M2 were taken from the authors' earlier study^65^. In their prior research, the authors presented an analytical framework aimed at estimating parameters for Windkessel-based outlet boundary conditions for intracranial arteries. This estimation is based on the geometrical characteristics of downstream arteries, which are discerned from clinical images. Utilizing the initial values from the authors' previous study for Windkessel parameters, including resistance and compliance, in the intracranial outlets simplified the iterative procedures. Otherwise, the process would have been laborious, time-intensive, and computationally demanding, requiring numerous iterations until alignment with clinical results was achieved. A few iterations were performed until results exhibited negligible variance, and these resistance values were recorded as subject-specific distal resistances for the branches of the intracranial arteries. It is crucial to highlight that, although the Windkessel model permits flexibility in resistance values, the necessity for the model to replicate precise clinical flow patterns introduces a distinctive aspect to the solution. Consequently, while having multiple sets of resistances is theoretically feasible, in reality, the set that concurrently aligns with clinical data is singular. Through this iterative calibration process, this study ensured that the resistance values we derived were not only statistically congruent with clinical measurements but also physiologically plausible, imparting a high degree of specificity to the validated model. A Windkessel-model based outlet boundary conditions were applied to all geometry outlets. The Windkessel model stands as a prominent conceptual framework within the realm of arterial blood flow simulations. It finds its application in the simulation studies of arterial blood flow dynamics. This model effectively integrates the distal vascular attributes such as distal vascular resistance as the outlet boundary conditions into the computational fluid dynamics (CFD) domain, as evidenced by prior research^3,11,66–75^. The Windkessel model is a lumped parameter construct aimed at emulating the hemodynamic aspects of the arterial network by emulating physiological correlations between vital hemodynamic parameters, including blood flow rate and pressure^73,76^.

The next objective of this investigation was to discern the most pivotal branches among ACA, M1, and M2, exerting a significant influence on ICA hemodynamics. This was achieved through the induction of stenosis within the distal branches of ACA, M1, and M2 by elevating distal resistances using the Windkessel model. The calculation of distal resistance corresponding to the level of distal stenosis was accomplished using the Hagen–Poiseuille equation (Eqs. 1, 2).

1 $$\Delta P=\frac{8\mu LQ}{\pi {r}^{4}}$$

2 $$R=\frac{8\mu L}{\pi {r}^{4}}$$

3 $$\Delta P=RQ$$

In this study, $\Delta P$ is the blood pressure drop along the conduit, $Q$ is the volumetric blood flow rate, $R$ is the distal resistance, $\mu$ is the blood viscosity, $L$ is the length of the artery and $r$ is the radius of the artery. For a constant, volumetric blood flow rate, blood pressure drop along the artery is directly proportional to the resistance in the artery (Eq. 3). This computation established a connection between the percentage change in pressure and the reduction in arterial diameter, as a response to an increased resistance to flow. This percentage change in the reduction of the arterial diameter represents the stenosis. Using this approach, the distal resistances were incrementally added to ACA, M1, and M2, simulating stenosis levels up to 60%. Stenosis was introduced individually to each branch to isolate its influence. The effect of stenosis in these branches was scrutinized by observing variations in pressure, velocity, and flow rate at specified points within the ICA, MCA, and ACA, in comparison to the baseline healthy artery condition. Additionally, Fractional Flow Reserve (FFR) analysis was integrated into the study. This evaluation aimed to gauge the impact of stenosis within M1, M2, and ACA on the distribution of blood pressure across the ICA and its associated branches. In the process of calculating FFR, the pre-stenotic pressure is derived by computing the area-weighted average pressure based on measurement planes situated before the stenotic segments. Conversely, the post-stenotic pressure is determined through analytical application of Hagen–Poiseuille equations (Eq. 3) since the stenosis is modelled at the outlets using the Windkessel model approach. By employing these approaches, this study aimed to identify the branches that wield the most considerable influence on hemodynamics within the ICA, thus contributing to a deeper comprehension of cerebrovascular health and the role of branch-specific stenosis in modulating blood flow dynamics.

### CFD simulation setup

**Figure 1:**
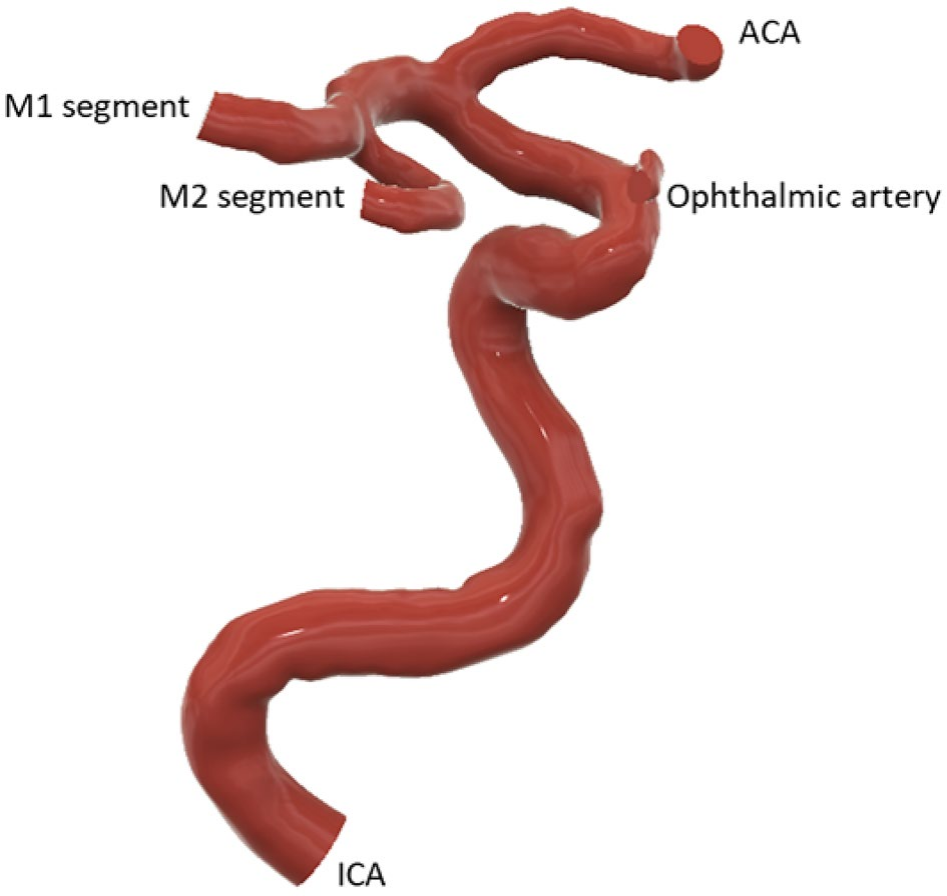
Subject specific geometry used in the CFD simulations.

**Figure 2:**
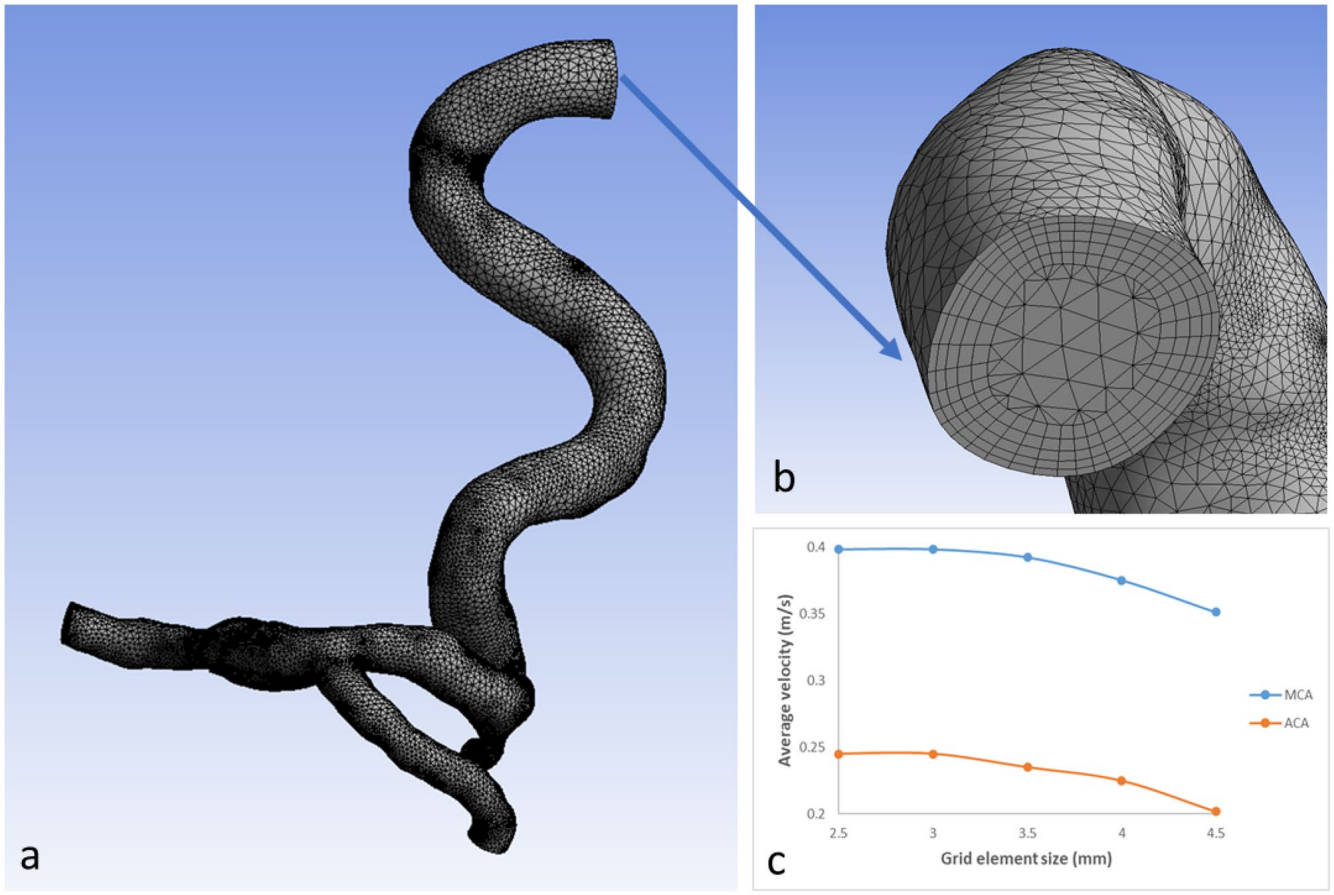
(**a**) Final mesh of subject specific geometry used in this study. (**b**) Wall refinement using inflation layers. (**c**) Mesh convergence analysis graph.

This study employed a subject-specific 3D model of the intra cranial arteries (ICA, OA, MCA and ACA) provided by See-Mode Technologies Pte Ltd (Fig. 1). The CFD simulations were conducted using the commercial software package ANSYS Fluent 2022 b. Meshing was carried out using Ansys Meshing in Workbench 2022b, employing tetrahedral grid elements with a minimum size of 0.3 mm. The resulting finer mesh comprised 177,594 nodes and 431,382 mesh elements, as determined through a mesh convergence study. To conduct the mesh convergence study, the grid element size was varied from a coarse mesh with grid elements ranging from 4.5 mm to a finer mesh with grid elements of 2.5 mm, and mesh convergence occurred with a grid element size of 3 mm. The average velocity at the MCA and ACA branches after the ICA bifurcation was utilized as the reference measurement for the mesh convergence study (Fig. 2). Refined meshing at wall locations was introduced into the simulation domain using inflation layers with a growth rate of 1.2 and a maximum wall thickness of 3 mm.

Blood flow within the model was considered laminar, incompressible and featuring non-Newtonian attributes which are the common assumptions used in the cerebrovascular blood flow modelling^77^. The non-Newtonian characteristics were incorporated using the Carreau model (Eq. 4), with specific parameter values: a Power-Law index ($n$)of 0.3568, time constant($\lambda$) of 3.313 s, Infinite Shear Viscosity(${\mu }_{\infty }$) of 0.0035 kg ms^−1^ and Zero Shear Viscosity (${\mu }_{0}$)of 0.056 kg ms^−1^. The density of blood flow was set at 1060 kg m^−3^.

4 $$\mu ={\mu }_{\infty }+\left({\mu }_{0}-{\mu }_{\infty }\right){[1+{\left(\lambda \gamma \right)}^{2}]}^{\frac{n-1}{2}}$$

**Figure 3:**
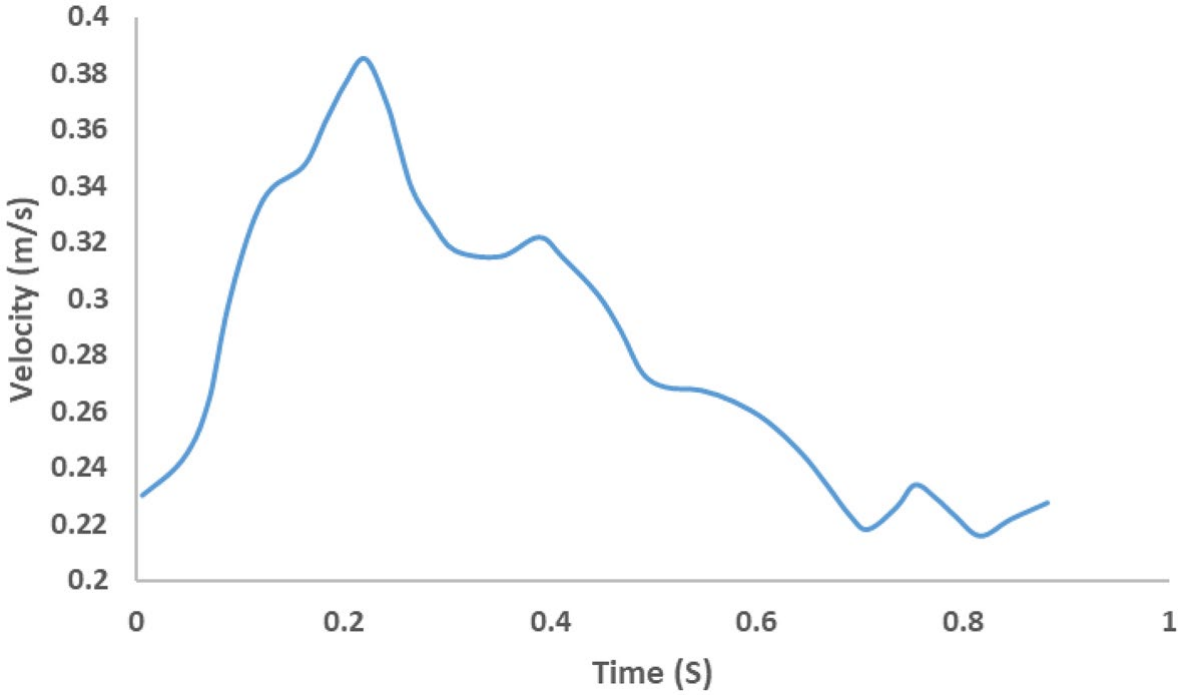
Inlet boundary conditions in CFD simulations derived from PCMRI data.

**Figure 4:**
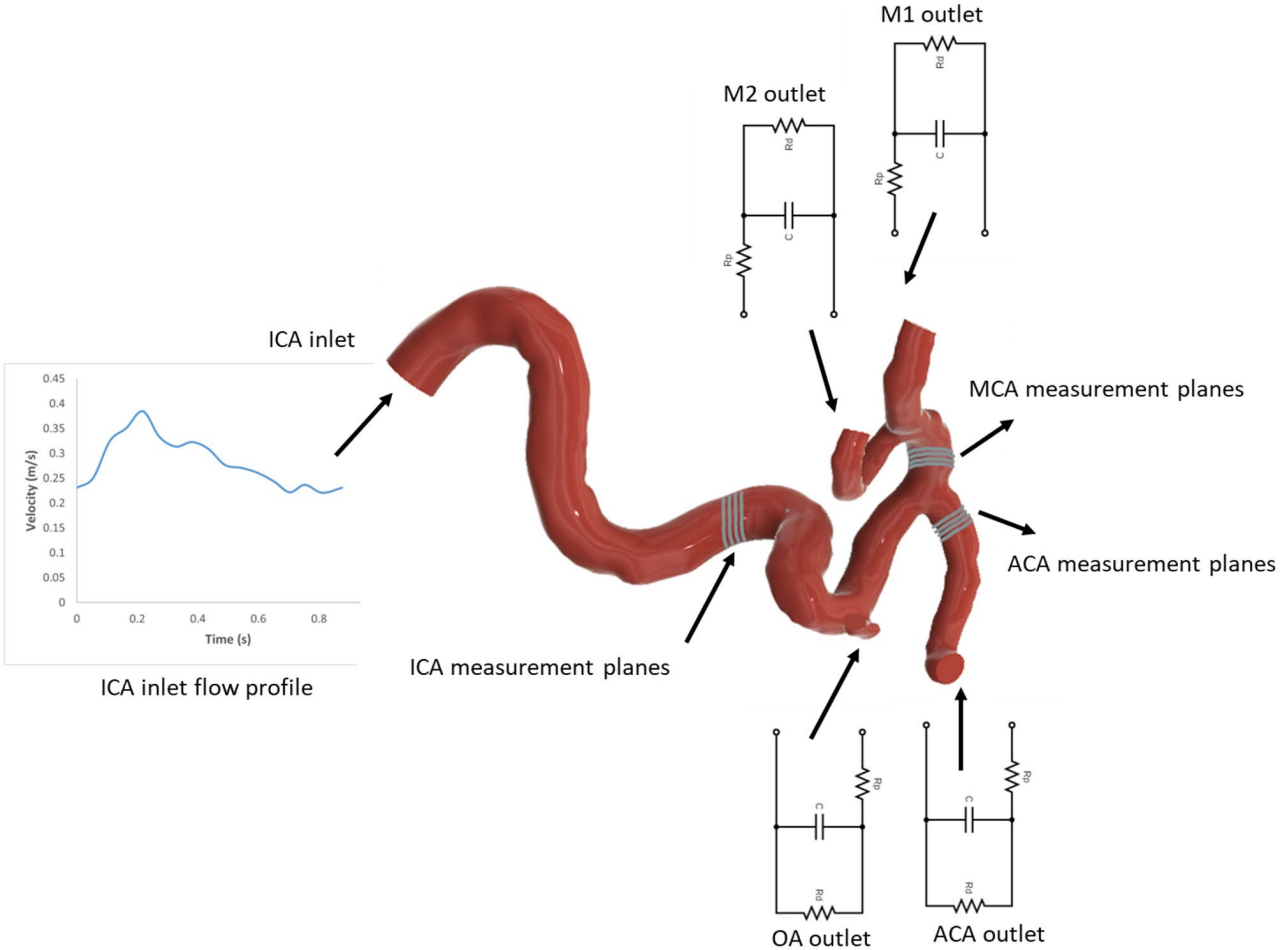
CFD model used for the subject specific intracranial arteries.

For the simulation of patient-specific blood flow, velocity measurements derived from internal carotid artery PCMRI data were implemented as the inlet boundary condition (Fig. 3). For the acquisition of time-resolved 4D flow MRI, a 3T MR scanner (MAGNETOM Skyra, Siemens, Erlangen, Germany) equipped with a 20-channel head/neck coil was employed. The imaging protocol focused on a healthy subject, encompassing vessels and the Circle of Willis (CoW), specifically targeting the basilar artery (BA) and bilateral intracranial internal carotid arteries, middle cerebral arteries, anterior cerebral arteries and posterior cerebral arteries (PCA). The scan parameters included a matrix size of 176 × 90, voxel dimensions of 0.5114 × 0.5114 × 1.2 mm, a repetition time (TR) of 57.72 ms, echo time (TE) of 3.38 ms, velocity encoding (VENC) of 100 cm s^−1^, and a flip angle of 15°. The imaging covered 416 slices over 13 cardiac cycles, with an acquisition average of 2 and a scan time ranging from 10 to 15 min. This comprehensive flow measurement workflow aimed to provide detailed insights into the dynamic aspects of blood flow within the specified vascular territories. This inlet blood flow rate was incorporated into the CFD domain using a User Defined Function (UDF). Outlet boundary conditions, encompassing M1, M2, and ACA, were defined based on analytically determined resistance and compliance values, as outlined in Tables 1. Figure 4 shows the representation of the CFD model used in this study.

**Table 1:** Resistance values corresponding to stenosis in the branches.

Stenosis (%)	Resistance (kg m^−4^ s^−1^)		
	M1	M2	ACA
0 (healthy case)	1.04E+08	2.43E+08	3.34E+08
10	1.59E+08	3.71E+08	5.08E+08
20	2.54E+08	5.94E+08	8.14E+08
30	4.34E+08	1.01E+09	1.39E+09
40	8.04E+08	1.88E+09	2.57E+09
50	1.67E+09	3.89E+09	5.34E+09
60	4.07E+09	9.50E+09	1.30E+10

The Simulation duration for one cardiac cycle was determined to be 0.876 s, derived from PCMRI data. The initial time step size of 0.01 s is determined using the Courant-Friedrichs-Lewy (CFL) number, a dimensionless parameter linking the time step size to the spatial grid size and fluid speed. In this study, the time step size is selected based on the maximum velocity of blood flow into the grid element, derived from PCMRI data, and the minimum grid element size determined from geometry meshing details. The simulation setup is assigned a Courant number of 1. The time step size was limited to 10^−4^ s to restrict further refinement of the simulation. The simulation employed an adaptive approach, with a solution convergence threshold of 10^−6^ for all variables. The simulation spanned 5 cardiac cycles. Through this organized CFD simulation setup, a comprehensive understanding of hemodynamic characteristics in the intracranial was achieved, enabling insightful insights into cerebrovascular health.

## Results

### CFD model validation

**Figure 5:**
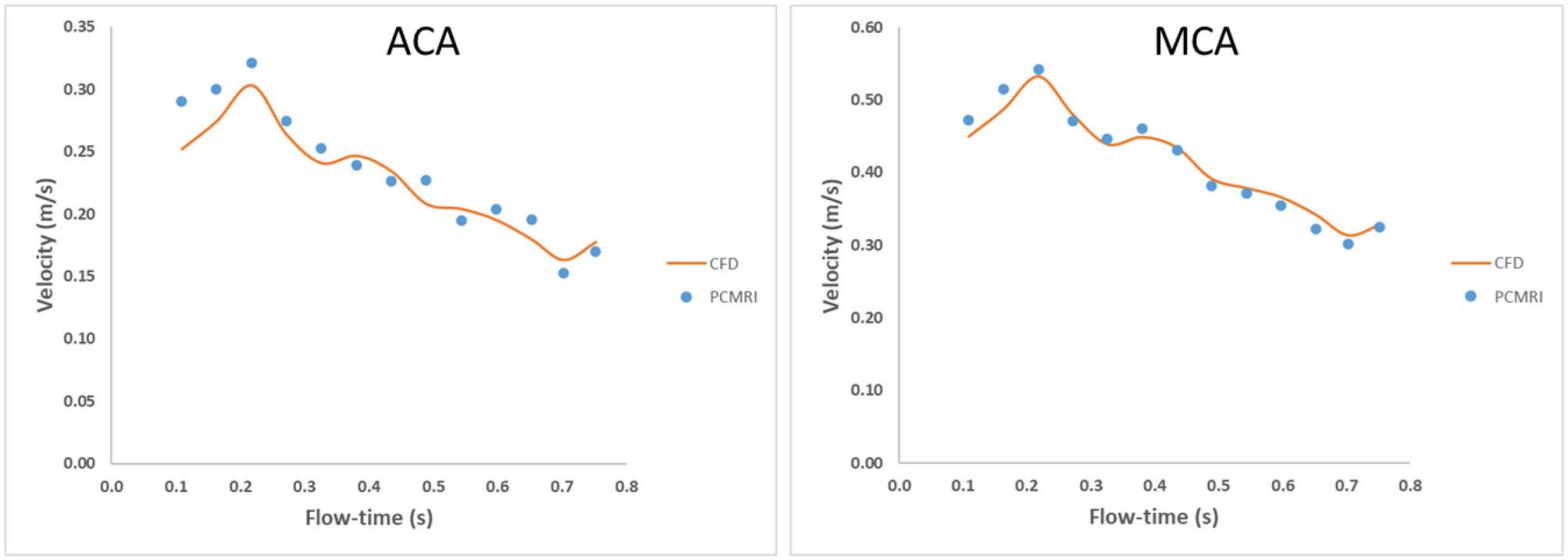
Validation of CFD results at ACA and MCA segments.

Before proceeding with further subject specific CFD simulations, it is imperative to validate the CFD model against clinical data. In this study, validation of the subject-specific CFD model was accomplished by utilizing Phase-Contrast Magnetic Resonance Imaging (PCMRI) data, presenting the averaged blood flow velocity measured across distinct adjacent planes within the MCA and ACA branches immediately after the bifurcation of the ICA into MCA and ACA. Figures 4 and 5 illustrate the validation process undertaken for this subject-specific CFD model.

For the validation process, the outlet resistances assigned to ACA, M1, and M2 through the Windkessel model were meticulously tuned. This was achieved to ensure that the averaged blood flow velocity from PCMRI clinical data coincided with the results of the CFD simulations. The CFD model incorporated the same planes used in PCMRI measurements to compute the average blood flow velocity, aligning the data for comparison. Given the utilization of a healthy geometry in this study, the resistance values derived from tuning outlets such as M1, M2, ACA, and OA were are 1.04E + 08 kg m^−4^ s^−1^, 2.43E + 08 kg m^−4^ s^−1^, 3.34E + 08 kg m^−4^ s^−1^ and 1.33e + 12 kg m^−4^ s^−1^ respectively .These resistance values were established as the outlets' resistance for the healthy case.

**Figure 6:**
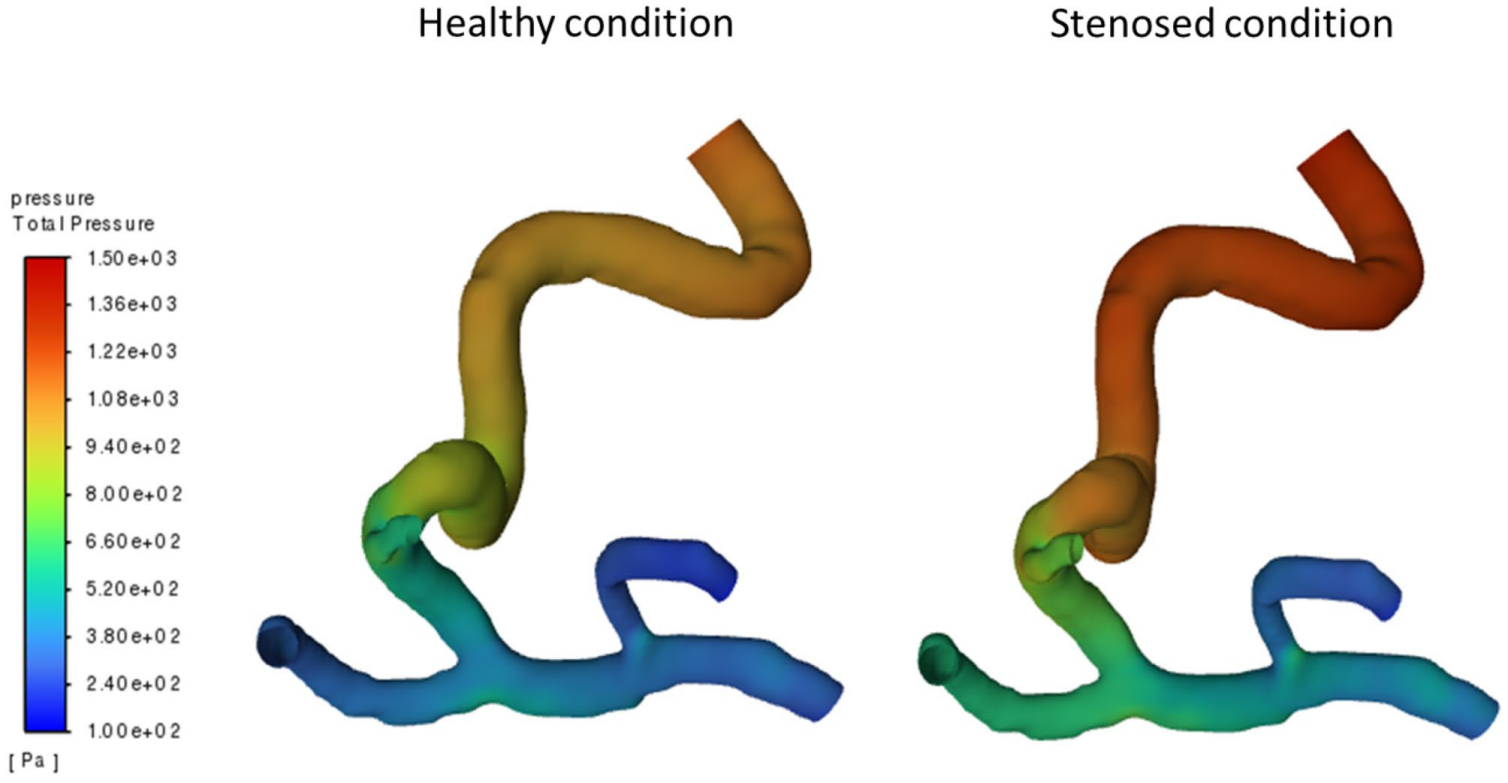
Pressure contours of the healthy case and stenosed case.

Figure 5 illustrates the distribution of average blood flow velocity within the ACA and MCA branches for both CFD and PCMRI results. For ACA branch, while minor variations emerge at the onset of the cardiac cycle, CFD results align with PCMRI data thereafter. In this case, the error percentage of the maximum blood flow velocity in the systolic phase is 5.7% where as the error percentage of the minimum velocity in the diastolic phase is 7%. For MCA branch, The congruence between CFD and PCMRI outcomes holds throughout the cardiac cycle. In this case, the error percentage of the maximum blood flow velocity in the systolic phase is 1.7% where as the error percentage of the minimum velocity in the diastolic phase is 4.2%. Given the successful validation demonstrated by Fig. 5, the CFD model is deemed accurate for the healthy case. With this validated CFD model, the incorporation of distal stenosis becomes feasible. This is achieved by applying distal resistance through the Windkessel model, with resistance values aligned with the corresponding stenosis levels. This approach enables the study to delve into the effects of distal stenosis on hemodynamics, further enriching our understanding of cerebrovascular health. Figure 6 presents a spatiotemporal illustration elucidating blood pressure dynamics across the arterial geometry in both a healthy scenario and a case with 60% stenosis. This depiction underscores the heightened capacity of CFD simulations to yield blood flow insights with superior spatial and temporal resolutions in contrast to conventional diagnostic instruments. Within Fig. 6, pressure contours are shown along the arterial geometry of a healthy scenario and a 60% stenosis in the ACA branch, revealing discernible distinctions in pressure distribution along the arterial pathway. This pressure differential manifests consistently throughout the entire arterial geometry. Specifically, the minimum pressure drop identified in the M2 segment region in the healthy scenario is 16.93 times less than that observed in the stenosed case at the same location. Simultaneously, the maximum pressure drop detected in the ICA region in the healthy scenario is 13.45 Pa less than the corresponding value in the stenosed case at the same location.

### Blood pressure variations at ICA, MCA and ACA

**Figure 7:**
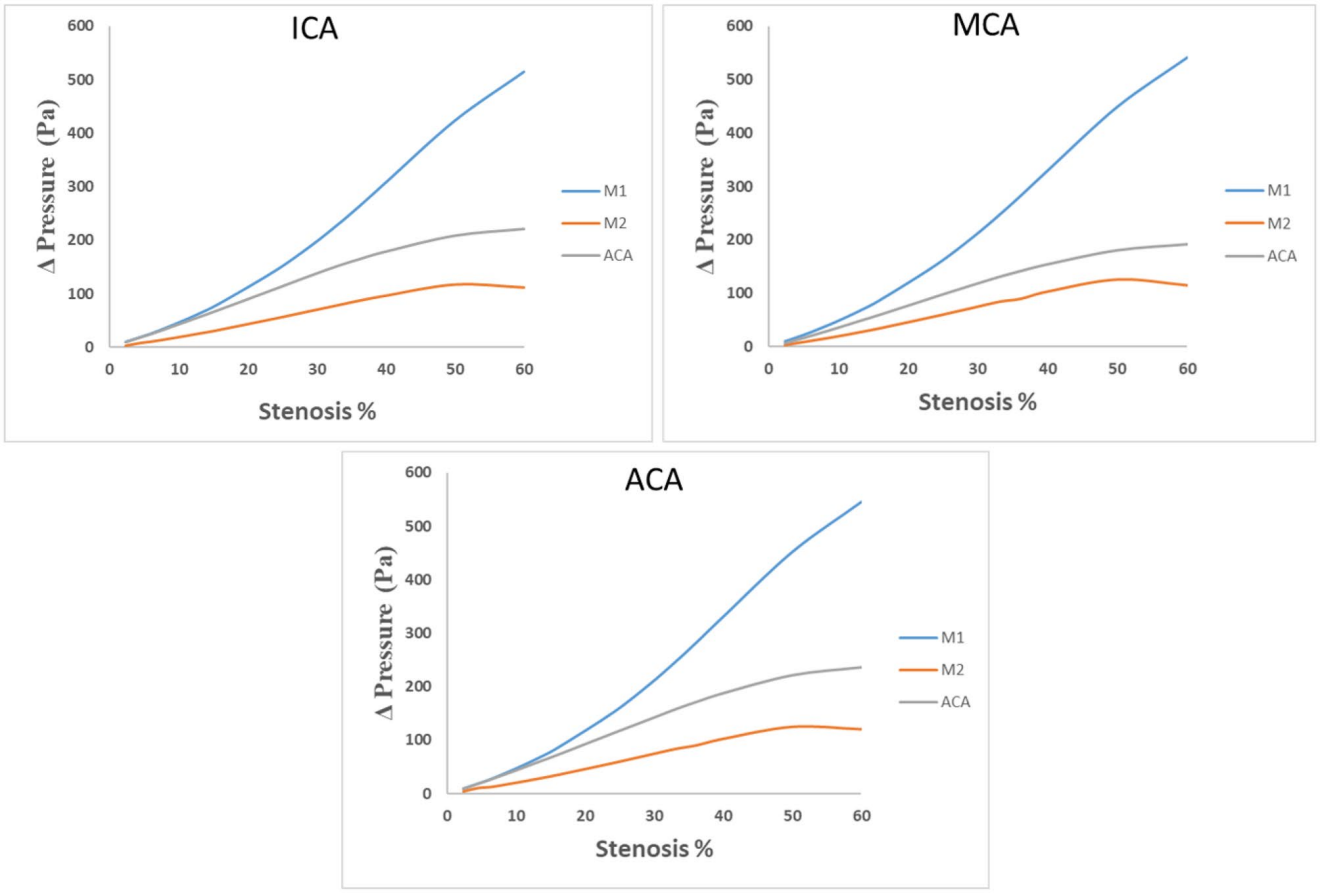
Pressure variations at ICA, MCA and ACA for the stenosis at M1, M2 and ACA.

Providing a comprehensive overview of blood pressure fluctuations within the ICA, MCA and ACA concerning stenosis in the M1, M2, and ACA branches, the data in Fig. 7 clearly demonstrates distinct patterns. For the ICA branch, the stenosis in the M1 segment demonstrates the most pronounced pressure variations, surpassing those observed in both the ACA and M2 branches. Particularly noteworthy is the substantial pressure decline, most notably evident with 60% stenosis. At this stenosis level, the pressure reductions experienced by the M1, ACA, and M2 branches are 514.88 Pa, 220.98 Pa, and 112.03 Pa, respectively. These findings underscore the pronounced impact of stenosis on the M1 branch. In the comparison of blood pressure variations at the ICA for ACA and M2 with those related to M1, the sensitivity to blood pressure changes caused by M1 stenosis was found to be 57.1% lower in the ACA branch, and the M2 branch displayed 78.2% less sensitivity to such variations.

In the MCA branch, the stenosis in the M1 segment consistently stands out with the most significant blood pressure fluctuations. Noteworthy is the M1 branch's highest pressure reduction at 60% stenosis, measuring 542.50 Pa at the MCA site, compared to the other branches. After M1, ACA experiences a considerable pressure decrease of 191.96 Pa at 60% stenosis, while M2 shows relatively milder blood pressure shifts, indicating a maximum decline of 115.30 Pa at the same stenosis level. Specifically, when examining blood pressure variations at the MCA, stenosis in the ACA branch reveals 64.61% lower sensitivity compared to the M1 branch. Similarly, stenosis in the M2 branch demonstrates 78.74% lower sensitivity than the M1 branch.

Analogously to the previous cases in the ICA and MCA branches, in the ACA branch, the stenosis in M1 continues to exhibit the most prominent pressure variations, followed by stenosis in ACA and M2. Once again, the most substantial pressure reduction is observed at 60% stenosis. For this condition, M1, ACA, and M2 branches undergo pressure decreases of 545.91 Pa, 236.36 Pa, and 120.18 Pa respectively, underscoring the pre-eminence of M1 branch in inducing pressure changes. When considering blood pressure variations at the ACA, stenosis in the ACA branch exhibited a sensitivity that was 56.71% lower than that of the M1 branch. Likewise, stenosis in the M2 branch displayed a sensitivity that was 77.98% lower than that of the M1 branch.

The data presented in the Fig. 7 underscores the pronounced impact of stenosis on blood pressure variations within different branches, with M1 consistently exerting the most substantial influence, followed by ACA and M2, as the degree of stenosis escalates. Through this meticulous examination of blood pressure variations, the distinct impacts of stenosis in different branches on blood pressure dynamics within the cerebrovascular system can be elucidated. This thorough analysis enhances the comprehension of the intricate relationship between stenosis and hemodynamics in cerebrovascular health.

**Figure 8:**
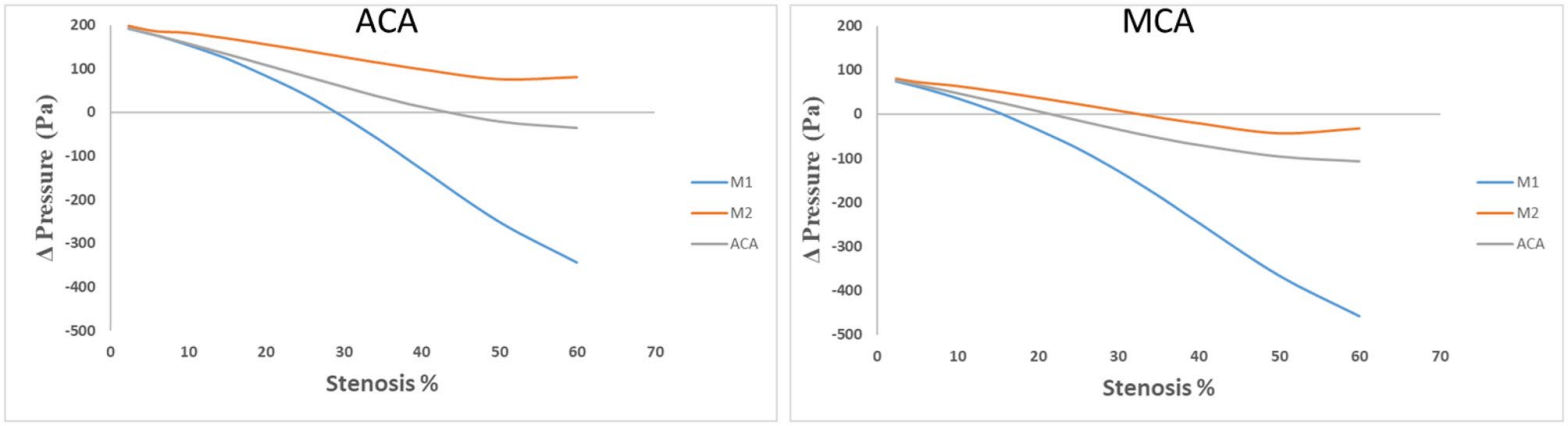
Pressure variations between ICA with respect to ACA and MCA for the stenosis at M1, M2 and ACA.

Figure 8 depicts the blood pressure variations between ICA and ACA, as well as between ICA and MCA, resulting from stenosis occurring in the M1, M2, and ACA branches. For the blood pressure variations between ICA and MCA, the data reveals distinct patterns, showcasing the substantial influence of stenosis on pressure gradients and the potential for reverse flow between these regions. Particularly noteworthy is the effect of M1 branch stenosis, which elicits the highest pressure drop variation. A remarkable maximum negative pressure gradient of 532.884 Pa is observed, signifying a heightened likelihood of reverse flow from the MCA to ICA for stenosis levels exceeding 15.9% within the M1 branch. Post the M1 stenosis scenario, the ACA branch demonstrates the most significant pressure variation. A maximum negative pressure gradient of 183.831 Pa is recorded, indicating a notable probability of reverse flow from the MCA to ICA for any stenosis surpassing 24% in the ACA branch. Conversely, stenosis within the M2 branch yields the least pressure variation, manifesting a maximum negative pressure gradient of 111.884 Pa. Reverse flow from the MCA to ICA is only anticipated after stenosis surpasses 33% in the M2 branch.

For the blood pressure variations between ICA and ACA, the data underscores the role of M1 branch stenosis, revealing the most substantial pressure drop variation. A noteworthy maximum negative pressure gradient of 536.18 Pa suggests a heightened potential for reverse flow from the ACA to ICA for stenosis levels exceeding 29.2% within the M1 branch. Subsequent to M1 stenosis, the ACA branch exhibits the most pronounced pressure variation, manifesting a maximum negative pressure gradient of 227.11 Pa. This points towards a significant likelihood of reverse flow from the ACA to ICA for any stenosis surpassing 40% in the ACA branch. Meanwhile, stenosis within the M2 branch yields a minimal pressure variation of 116.46 Pa, with no discernible evidence of reverse flow from ACA to ICA for stenosis levels up to 60% within the M2 branch.

By delving into pressure gradient variations and potential reverse flow scenarios, Fig. 8 provide crucial insights into the complex hemodynamic interplay within the cerebrovascular system. This analysis further contributes to the understanding of how stenosis in various branches can lead to alterations in pressure dynamics and the possibility of reverse flow, significantly impacting cerebrovascular health.

### Average blood flow velocity and flow rate

**Figure 9:**
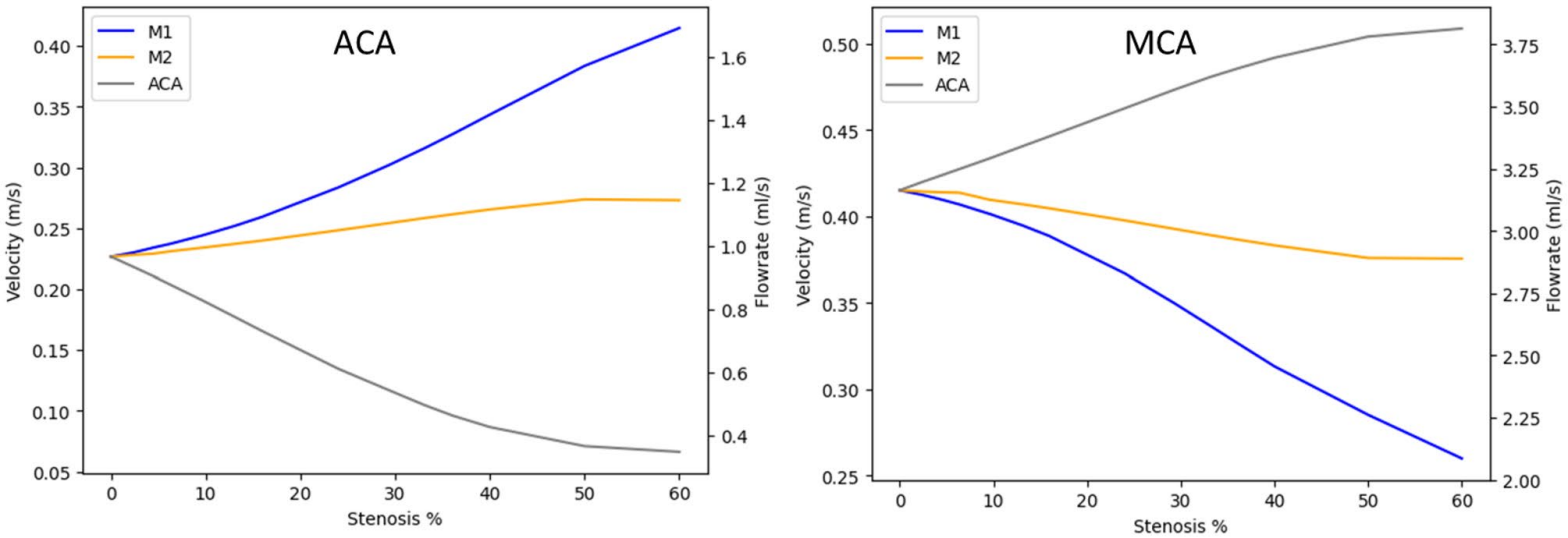
Average blood flow velocity and flow rate in the ACA and MCA branches for the stenosis at M1, M2 and ACA.

A comprehensive evaluation of the area-weighted average of blood flow velocity and blood flow rate within the ACA and MCA branch under stenotic conditions in the M1, M2, and ACA branches is shown in the Fig. 9. For ACA location, Stenosis within the M1 branch leads to a substantial increase in average velocity, escalating from 0.226 to 0.414 m s^−1^ as stenosis increases up to 60%. This translates to a velocity increment of 0.188 m s^−1^. In contrast, stenosis within the ACA branch yields a marked decrease in average velocity, dropping from 0.226 to 0.066 m s^−1^ as stenosis increases up to 60%. This indicates a velocity drop of 0.160 m s^−1^. Stenosis in the M2 branch demonstrates relatively minor variations in average velocity within the ACA branch compared to stenosis in the other two branches. Notably, a slight velocity increment of around 0.046 m s^−1^ is observed for M2 branch stenosis as it increases up to 60%. At MCA location, specifically, stenosis within the M1 branch results in a notable decrease in average velocity, with a reduction from 0.415 to 0.259 m s^−1^ observed as stenosis increases to 60%. This accounts for a velocity drop of 0.155 m s^−1^. Conversely, stenosis in the ACA branch leads to an increase in average velocity, rising from 0.415 to 0.508 m s^−1^ for a stenosis increase of up to 60%, representing a velocity increment of 0.093 m s^−1^. Notably, stenosis within the M2 branch shows relatively minimal variations in average velocity within the MCA branch compared to stenosis in the other two branches. The recorded velocity drop for M2 branch stenosis is approximately 0.039 m s^−1^ as stenosis increases up to 60%.

At ACA location, Stenosis within the M1 branch leads to a significant increase in average flowrate, escalating from 0.964 to 1.880 ml s^−1^ as stenosis increases up to 60%. This accounts for a flowrate increment of 0.916 ml s^−1^. In contrast, stenosis within the ACA branch results in a marked decrease in average flowrate, dropping from 0.964 to 0.082 ml s^−1^ as stenosis increases up to 60%. This indicates a flowrate drop of 0.882 ml s^−1^. Stenosis in the M2 branch demonstrates relatively minor variations in average flowrate within the ACA branch compared to stenosis in the other two branches. A slight flowrate increment of around 0.219 ml s^−1^ is observed for M2 branch stenosis as it increases up to 60%. At MCA location Specifically, stenosis within the M1 branch results in a substantial decrease in average flowrate, with a reduction from 3.206 to 1.988 ml s^−1^ observed as stenosis increases up to 60%. This corresponds to a flowrate drop of 1.218 ml s^−1^. Conversely, stenosis in the ACA branch leads to a notable increase in average flowrate, rising from 3.206 to 3.941 ml s^−1^ as stenosis increases up to 60%, representing a flowrate increment of 0.734 ml s^−1^. Notably, stenosis within the M2 branch shows relatively minor variations in average flowrate within the MCA branch compared to stenosis in the other two branches. A slight flowrate drop of about 0.299 ml s^−1^ is observed for M2 branch stenosis as it increases up to 60%. Figure 9 provide valuable insights into the intricate relationship between stenosis in specific branches and the resultant alterations in area-weighted average of blood velocity and flowrates within both the MCA and ACA branches. These analyses contribute to an enhanced comprehension of how stenosis-induced changes in flowrates can impact the overall hemodynamic behaviour in these critical cerebrovascular regions.

**Figure 10:**
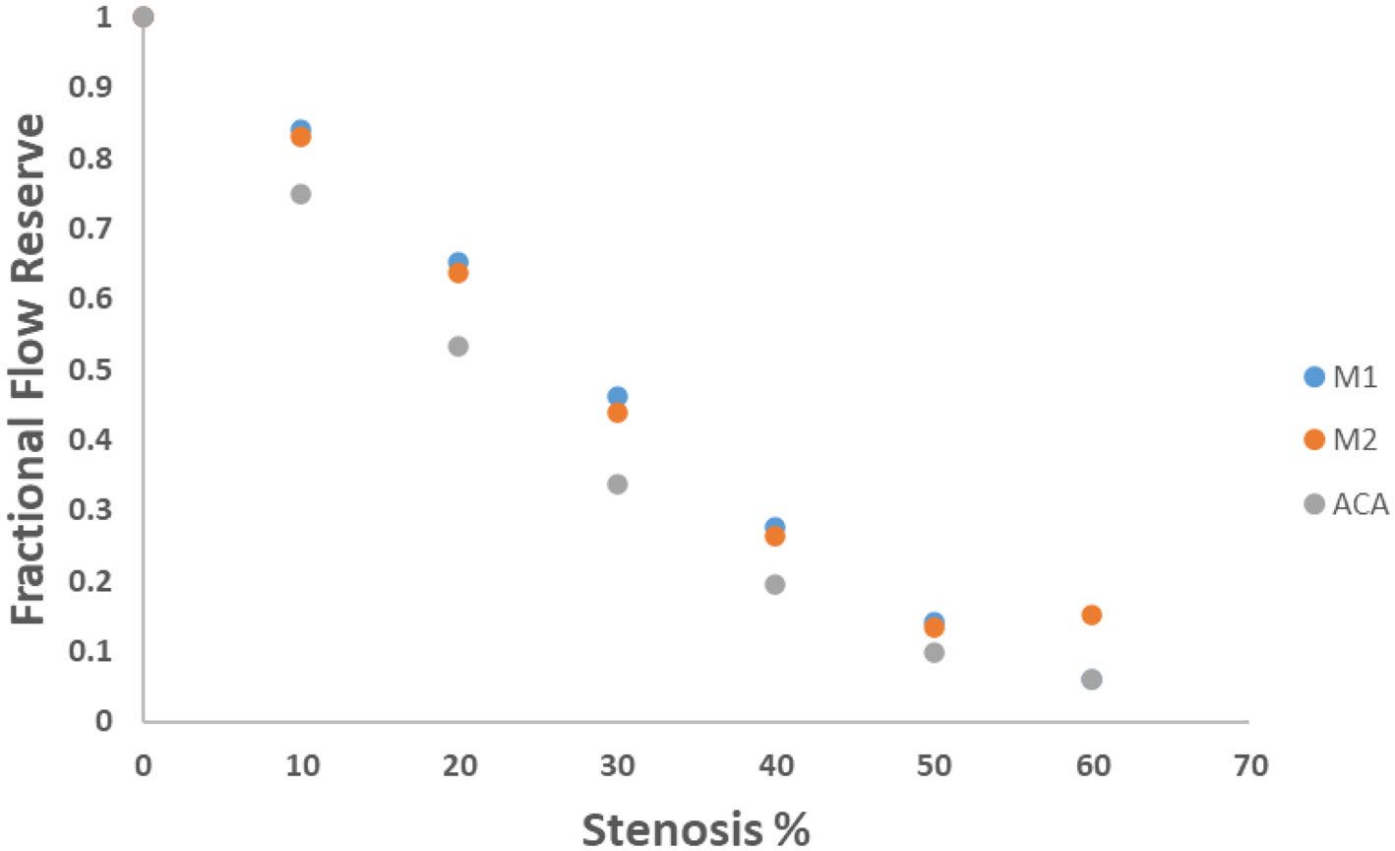
Fractional Flow Reserve for increase in stenosis at branches of M1, M2 and ACA.

Fractional flow reserve (FFR) serves as a targeted indicator of the severity of stenosis within a specific lesion. It quantifies the ratio of the maximum flow in the presence of a stenosis to the normal maximum flow. Figure 10 presents valuable insights into the FFR values for various degrees of stenosis in the M1, M2, and ACA branches of the internal carotid artery. As the degree of stenosis increases, FFR values progressively decrease, reflecting the diminishing capacity for blood flow. Specifically, For the M1 branch, the FFR rate decreases from 0.84 for a 10% stenosis to less than 0.1 for a 60% stenosis. In the case of the M2 branch, the FFR rate undergoes a decrease from 0.83 for a 10% stenosis to 0.15 for a 60% stenosis. Within the ACA branch, the FFR rate experiences a reduction from 0.75 for a 10% stenosis to less than 0.1 for a 60% stenosis. These data signifies that there is substantial reduction in the flow capacity as the stenosis becomes more severe. Figure 10 offers a clear representation of how FFR values change in response to varying degrees of stenosis in the different branches of the internal carotid artery. This analysis underscores the role of FFR in providing a quantitative measure of the impact of stenosis on flow capacity, aiding in the assessment of lesion severity and potential treatment decisions.

## Discussion

The accuracy of the CFD model plays a pivotal role in predicting the most realistic flow patterns within the carotid artery bifurcation. It enables us to uncover the precise interplay between various hemodynamic factors that contribute to the initiation, progression, and development of atherosclerosis^78^. However, studying the hemodynamics of carotid arteries using CFD faces challenges due to the complex biological system and individual variations driven by genetics, making numerical investigations intricate. Hence, a well-crafted numerical simulation that effectively captures actual blood flow physics becomes essential for generating authentic simulation outcomes^79^. In this research, a comprehensive patient-specific data, including individual geometry and personalized inlet blood flow conditions is utilized to develop the CFD model. The validation of this model against clinical results, as depicted in Fig. 5, ensures its accuracy. A notable advantage of employing CFD simulations is the ability to obtain blood flow results with higher spatial and temporal resolutions compared to conventional diagnostic tools^80^.

This study focused on investigating blood pressure variations within the internal carotid artery (ICA) and its branches, including M1, M2, and ACA, under different stenosis scenarios. Blood pressure fluctuations serve as crucial indicators for assessing the severity of atherosclerosis, as documented in various studies. For instance, research by Nitesh Kumar et al. highlighted that significant blood pressure fluctuations during the cardiac cycle lead to arterial wall deformations, particularly during peak systole. This deformation prompts arterial enlargement, resulting in altered wall shear stress patterns and the formation of recirculation zones^2^. As outlined in the introduction, despite the growing popularity of integrating fluid–structure interaction (FSI) studies into Computational Fluid Dynamics (CFD) models for exploring arterial wall flexibility, this study utilized rigid wall assumptions as the primary focus here is on estimating blood pressure drop and Fractional Flow Reserve (FFR) at different locations within the intracranial internal carotid artery. This choice is motivated by the minimal impact of blood pressure variations under rigid wall assumptions and the high computational cost associated with FSI models.

In this study, when examined blood pressure variations at the internal carotid artery (ICA) in response to increasing stenosis levels in the M1, M2, and ACA branches (as shown in Fig. 6), it is observed a distinct sensitivity among these branches. Specifically, the M1 branch demonstrated a high sensitivity to blood pressure variations at the ICA corresponding to stenosis increases in the M1 branch. Following M1, the ACA branch exhibited the next level of sensitivity. This implies that any stenosis occurring in the M1 branch would likely be detectable through variations in blood pressure at the ICA. On the contrary, the M2 branch exhibited minimal sensitivity to blood pressure variations at the ICA resulting from stenosis. When comparing the relative blood pressure variations at the ICA for ACA and M2 with those for M1, it is found that the ACA branch had 57.1% less blood pressure variation sensitivity, while the M2 branch displayed 78.2% less sensitivity compared to the blood pressure variations caused by M1 stenosis. This observation is crucial, indicating that relying solely on monitoring blood pressure variations at the ICA may not adequately serve as an indicator for detecting stenosis in the M2 branch. However, it's important to note that the presence of stenosis in the M2 branch can lead to significant disruptions in cerebral blood flow beyond the M2 branch. These disruptions can have serious consequences for the patient, potentially leading to strokes or other severe complications. Thus, while blood pressure variations at the ICA can provide valuable insights, a comprehensive approach that takes into account a wider range of hemodynamic factors and potential consequences is essential for accurate diagnosis and patient care.

When analysed blood pressure variations at both the anterior cerebral artery (ACA) and the middle cerebral artery (MCA) for various levels of stenosis in the M1, M2, and ACA branches, it is observed a consistent trend in which The M1 branch exhibited high sensitivity to blood pressure variations at both ACA and MCA, while the M2 branch displayed lower sensitivity to these variations. Specifically, when looking at blood pressure variations at the MCA, stenosis in the ACA branch showed a sensitivity that was 64.61% lower than that of the M1 branch. Similarly, stenosis in the M2 branch displayed a sensitivity that was 78.74% lower than that of the M1 branch. When considering blood pressure variations at the ACA, stenosis in the ACA branch exhibited a sensitivity that was 56.71% lower than that of the M1 branch. Likewise, stenosis in the M2 branch displayed a sensitivity that was 77.98% lower than that of the M1 branch. These findings further underscore the varying sensitivities of different branches to blood pressure variations, with the M1 branch being highly sensitive and the M2 branch being less sensitive. This emphasizes the importance of considering branch-specific responses when interpreting blood pressure variations and their implications for potential stenosis.

As elucidated in the results section, the analysis of blood pressure variations between the anterior cerebral artery (ACA) and the middle cerebral artery (MCA), in comparison to blood pressure at the internal carotid artery (ICA), has provided valuable insights into the likelihood of significant reverse flow within different arterial locations for varying percentages of stenosis in the M1, M2, and ACA branches. The findings indicate that the potential for reverse flow can be identified at an earlier stage in cases of stenosis at the M1 branch. The onset of reverse flow can be detected from a stenosis percentage as low as 15.9% in the M1 branch, when comparing blood pressure variations between ICA and MCA. However, the reverse flow is expected to be detectable from a stenosis percentage of 29.2% when analysing blood pressure variations between ICA and ACA. This underscores that analysing blood pressure variations between ICA and MCA serves as a more sensitive and early indicator of reverse flow compared to analysing blood pressure variations between ICA and ACA, even for stenosis in the ACA branch.

For stenosis in the ACA branch, the likelihood of reverse flow can be detected for stenosis percentages starting from 24%, as observed from the blood pressure variations between ICA and MCA. In contrast, the reverse flow is expected to be detectable from a stenosis percentage of 40% when analysing blood pressure variations between ICA and ACA. This reaffirms the importance of analysing blood pressure variations between ICA and MCA as a primary indicator for identifying chances of reverse flow within the internal carotid artery. In the case of stenosis in the M2 branch, the chance of reverse flow is discernible from a stenosis percentage of 33% when analysing blood pressure variations between MCA and ICA. However, no significant chance of reverse flow is observed for stenosis in the M2 branch when comparing blood pressure variations between ICA and ACA. This further underscores that blood pressure variations between ICA and ACA are a key indicator for identifying the potential for reverse flow within the internal carotid artery.

Additionally, when assessing the relative blood pressure variations between ICA and MCA, stenosis in the M1 branch exhibits higher sensitivity compared to stenosis in other branches. Stenosis at the ACA branch displays 65.51% less blood pressure variations compared to the M1 branch, while stenosis at the M2 branch exhibits 79% less blood pressure variations relative to the M1 branch. Similarly, when considering relative blood pressure variations between ICA and ACA, stenosis in the M1 branch demonstrates higher sensitivity compared to stenosis in other branches. Stenosis at the ACA branch shows 57.64% less blood pressure variations compared to the M1 branch, and stenosis at the M2 branch exhibits 78.27% less blood pressure variations relative to the M1 branch.

When stenosis is applied to the M1 branch, the blood velocity at MCA experiences a substantial drop of up to 37.59% for stenosis up to 60%. On the other hand, when stenosis is applied to the ACA branch, the blood velocity at MCA increases by 22.40%. This phenomenon indicates that stenosis in one branch can lead to changes in blood flow distribution within the artery, causing compensation mechanisms to come into play. The increase in blood velocity at the MCA could be a compensatory mechanism to counteract the blood flow restrictions caused by stenosis in the ACA branch. It’s noteworthy that the M2 branch remains less sensitive to changes in blood flow velocity, which is consistent with the previous observations of blood pressure variations. This could be due to the specific anatomical and hemodynamic characteristics of the M2 branch, making it less responsive to alterations in blood flow caused by stenosis in other branches.

When analysing the average blood flow velocity at the ACA branch for stenosis in the M1, M2, and ACA branches, a similar trend is observed as in the MCA. Stenosis in the M1 branch results in a significant increase in blood velocity at the ACA branch by 83%, while stenosis in the ACA branch itself leads to a drop in blood flow velocity by 70.79%. This further emphasizes the intricate relationship between different branches and their contributions to blood flow redistribution within the arterial system. These findings emphasize the intricate adjustments made by the cerebrovascular system to adapt to changes in blood flow due to stenosis in different branches. The compensation mechanisms involve altering blood velocity to maintain adequate perfusion to downstream regions, which underscores the complex nature of hemodynamic interactions in the arteries.

When stenosis is introduced in the M1 branch, the blood flow rate at the MCA experiences a reduction of up to 38% for stenosis up to 60%. This reduction indicates that the stenosis in the M1 branch has led to a significant decrease in the overall blood flow rate in the downstream MCA. On the other hand, when stenosis is applied to the ACA branch, the blood flow rate at MCA increases by 22.90%. This increase suggests that the cerebrovascular system is increasing the blood flow rate in the MCA to compensate for the reduced flow caused by the stenosis in the ACA branch. As mentioned before, similar to the trends observed with blood pressure and blood flow velocity, the M2 branch remains less sensitive in terms of blood flow rate changes. This consistent behaviour underscores the complex relationship between these hemodynamic factors within the vascular system. When examining the average blood flow rate at the ACA branch, the patterns mirror those observed at the MCA branch. Stenosis in the M1 branch leads to a significant increase in blood flow rate at the ACA by 95%, potentially as a compensatory response to the reduced flow caused by the stenosis. Conversely, stenosis in the ACA branch results in a substantial drop in blood flow rate at the ACA branch by 91.49%. This highlights how stenosis in one branch can indeed lead to significant alterations in blood flow distribution to maintain appropriate perfusion to various regions.

These findings emphasize the complex and interconnected nature of hemodynamic adjustments within the vascular system in response to stenosis. The observed trends of compensation in blood flow rate across different branches further underscore the body's efforts to ensure optimal blood flow despite the presence of arterial stenosis.

In the domain of cerebrovascular research, initial investigations have hinted at the potential of Fractional Flow Reserve (FFR) as a valuable predictor for recurrent stroke in patients with symptomatic intracranial arterial stenosis (ICAS)^81^. FFR holds promise in identifying ischemia specific to lesions, offering insights into the hemodynamic significance of ICAS. Therefore, understanding the factors that impact cerebral FFR becomes a pivotal pursuit^18,82–84^.

FFR value is calculated by dividing the post-stenotic pressure by the pre-stenotic pressure. This value is dimensionless, ranging from 0 to 1. In cardiovascular clinical application, an FFR value of 1.0 signifies normal blood flow beyond the stenosis, indicating no significant hemodynamic compromise. An FFR value approaching 0.80 or lower often serves as a threshold to identify hemodynamically significant coronary artery stenosis. A value of 0.80 or below suggests a notable reduction in blood flow across the stenosis, potentially warranting intervention like angioplasty or stent placement^85–89^. Within the cerebrovascular context, there is no established threshold value for FFR measurements when evaluating Intracranial Atherosclerotic Stenosis^48^. Nonetheless, in a particular study, a threshold value of FFR ≤ 0.70 was employed to determine the necessity for stenting in cases of hemodynamically significant stenosis. Additionally, the same study utilized FFR values ≤ 0.8 as a basis for determining the appropriate medical treatment approach for certain patients^90^. In this study, the examination of FFR values for stenosis in branches at M1, M2, and ACA revealed that FFR drops below 0.8 for stenosis exceeding 12.4% in the M1 branch, 11.8% in the M2 branch, and 7.7% in the ACA branch. The rate of FFR decrease for stenosis up to 60% was 94.1% in the M1 branch, 84.9% in the ACA branch, and 93.9% in the M2 branch. The impact of stenosis in the M2 branch becomes more evident through FFR compared to other factors like blood pressure variations, blood flow velocity, and blood flow rate. Thus, FFR proves to be a robust indicator for stenosis in the M2 branch.

This study has a significant limitation in that it employs a 0D Windkessel-based stenosis model without incorporating the physical effects of stenosis within the geometry. In addition, the geometry utilized in this research exclusively assesses blood flow in the right anterior branches, without accounting for collateral blood flow compensation mechanisms, including primary or leptomeningeal collaterals from the left cerebral hemisphere. These constraints may lead to variations in the range of FFR measurements obtained in this study compared to those in clinical investigations. Furthermore, in this investigation, rigid wall modelling is employed instead of integrating a fluid–structure interaction model, primarily due to computational cost considerations. Nonetheless, it is essential to recognize that fluid–structure interaction modelling plays a pivotal role in exploring the intricate dynamics of intracranial pathologies. This approach provides a more accurate approximation to in vivo conditions by accounting for the deformable cerebral vasculature's response to pulsatile flow.

## Conclusions

In conclusion, this study delved into the complex interplay of hemodynamic factors within the carotid artery bifurcation, shedding light on the implications of blood pressure variations, blood flow velocity, blood flow rate, and Fractional Flow Reserve (FFR) in the context of arterial stenosis. The challenges associated with characterizing the hemodynamics of carotid arteries through numerical simulations stem from the multifaceted nature of the biological system and the inherent individual variations. Our approach addressed these challenges by incorporating patient-specific data, including geometrical parameters and inlet blood flow conditions. The validation of our CFD model against clinical results attests to its accuracy, enhancing the reliability of our findings. This study revealed that different branches of the carotid artery exhibit varying sensitivities to blood pressure variations. The M1 branch displayed high sensitivity, followed by the ACA branch, while the M2 branch exhibited minimal sensitivity. This differential response underscores the need for a nuanced approach when interpreting blood pressure variations as indicators of potential stenosis. The comparison of blood pressure variations between different arterial locations—internal carotid artery (ICA), middle cerebral artery (MCA), and anterior cerebral artery (ACA)—unveiled valuable insights into the likelihood of reverse flow. Analysing blood pressure variations between ICA and MCA emerged as a more sensitive indicator of reverse flow compared to analysing variations between ICA and ACA. This highlights the significance of MCA in reflecting potential reverse flow conditions. The examination of blood flow velocity and blood flow rate under different stenosis scenarios showcased the intricate compensatory mechanisms within the cerebrovascular system. Stenosis in one branch prompted adjustments in blood flow distribution in other branches, underscoring the complex interplay of factors influencing blood perfusion. The introduction of FFR as an indicator further strengthened our analysis. FFR values dropped below the critical threshold of 0.8 for stenosis percentages above specific thresholds in different branches. FFR emerged as a promising predictor of stenosis severity, with its sensitivity particularly pronounced in the M2 branch.

## Data Availability

The datasets generated during and/or analysed during the current study are available from the corresponding author on reasonable request.
